# Evaluation of Mental Load of Drivers in Long Highway Tunnel Based on Electroencephalograph

**DOI:** 10.3389/fpsyg.2021.646406

**Published:** 2021-10-04

**Authors:** Yanqun Yang, Yang Feng, Said M. Easa, Xinyi Zheng

**Affiliations:** ^1^College of Civil Engineering, Fuzhou University, Fuzhou, China; ^2^Traffic Research Center, Fuzhou University, Fuzhou, China; ^3^Department of Civil Engineering, Ryerson University, Toronto, ON, Canada; ^4^Faculty of Humanities and Social Sciences, Fuzhou University, Fuzhou, China

**Keywords:** driver mental state, driving simulator, electroencephalogram, highway tunnel, car following

## Abstract

In recent years, the mileage of the tunnels has substantially increased with the rapid highway construction that led to increasing highway tunnels. Most studies on tunnel accidents have mainly focused on the external environments, such as tunnel structure, traffic volume, and lighting. In addition, although many studies on mental load of drivers have been conducted for public roads, such studies for highway tunnels have been limited. In this study, three scenarios with different front vehicle speeds (60, 45, and 30 km/h) in a two-lane long tunnel (one lane in each travel direction) were evaluated using a driving simulator. The experiment involved 24 participants (14 men and 10 women) with an average age of 25.8 years and an average experience of 3.2 years. The electroencephalogram (EEG) technology was used to collect the leading EEG indicators during the driving simulation of the scenarios: α, β, and θ waves and the wave ratio, (α + θ)/β. According to the β-wave energy measurements, the alertness of drivers was the lowest at 45 km/h after adapting to the tunnel environment, indicating that the drivers were more comfortable at this speed. This preliminary finding should help in determining the speed limit in this type of tunnel.

## Introduction

The tunnel is an alternative solution for the roadway along rugged topography to overcome natural conditions. Thus, road tunnels usually represent bottlenecks in the road network (Bassan, 2016). The tunnels are at risk of hazard and intractable accidents. The severe accident rates and costs in tunnels were often higher than those on the corresponding roads (Caliendo and De Guglielmo, 2012). Therefore, safety studies on driving in tunnels are necessary.

For general highways, many studies on mental load of drivers have been conducted using electroencephalograph (EEG). These studies have made it possible to evaluate mental load and behavior of drivers in tunnels. The studies have used different indicators, such as heart rate variability, eye-tracking movements (Yang et al., 2019b, 2020d), and EEG. Using EEG to study the mental state of drivers in a driving simulator is a mature technique, which was first used in the 1980's (Lemke, 1982; Torsvall, 1987). The accuracy and efficiency of this technology have been demonstrated (Haak et al., 2008; Li et al., 2010; Borghini et al., 2012; Bashivan et al., 2016; Kim et al., 2018). Many researchers have studied essential driver characteristics, such as driver sleepiness (Resalat and Saba, 2015), drowsiness (Lin et al., 2005), fatigue (Jap et al., 2009; Arakawa et al., 2019), alertness level (Kiymik et al., 2004), and cognitive load (Barua et al., 2017). Other studies have used EEG, as the effect of color scheme (Yang et al., 2020a), situation awareness (Yang et al., 2020b), and the effect of directional signs (Yang et al., 2020c). Other studies on brain activities, driver aggressiveness, music and emotion, and EEG classification have also been conducted (Fan et al., 2010; Liu et al., 2013; Lin et al., 2014; Yang et al., 2018, 2019a; Zeng et al., 2018).

For tunnels, numerous studies have been conducted on traffic safety. However, most studies have focused on tunnel geometric characteristics and traffic volumes, tunnel illumination and visibility (Miyake et al., 2019), accident zone locations (Amundsen and Ranes, 2000; Ma et al., 2009), and safety speed (Yan et al., 2019). The interesting study by Yan et al. (2019) addressed driving risk levels in continuous tunnels, 250–1,000 m long and two lanes in each direction, using various risk indicators. A naturalistic driving system equipped with a road environment and driving-behavior data acquisition system was used to collect the data in 130 tunnels on four highways. The American Association of State Highway and Transportation Officials (AASHTO) braking model and the convex hull algorithm were used to predict the critical safety speed and the critical time headway of each risk feature. In contrast, this study was designed to predict the speed of car-following that is most comfortable for the drivers using brain waves and eye movements. Specifically, this study evaluated the effect of a 1,800-m long tunnel on the EEG of drivers in a car-following situation. Thus, this study complements the study by Yan et al. (2019).

However, a few studies have been conducted on driver behavior in tunnels. The literature shows that the pathological discomfort of drivers strongly correlates with the accident rate when driving in tunnels (Calvi and D'amico, 2013). The behavioral training using virtual reality affects the self-evacuation during a drill (Kinateder et al., 2013). Thus, more studies on driver behavior in tunnels are needed. The novelty of this study lies in the evaluation of the mental state of drivers to determine the appropriate speed limit in a highway tunnel, which has not been addressed in the literature. In the experimental scenarios, the driver followed a car in front, which traveled at a specific speed to simulate different speed limits. The EEG of drivers was measured to determine the change in the mental state of drivers, mainly those undesirable emotions, such as tension, depression, and anger. The findings of this study might strengthen the inference and analysis of the motivation and mechanism of the lousy behavior of drivers and might provide a theoretical basis for the prevention of tunnel traffic accidents.

## Experimental Design

### Experimental Subjects

Drivers of similar ages were selected to avoid the influence of age on driving. A total of 24 subjects were recruited for the experiments (14 men and 10 women). Each participant was in good physical condition, with no color weakness or color blindness. The driving experience of the subjects varied. The average age was 25.8 years, with an SD of 5.5 years. The average experience was 3.2 years, with an SD of 3.7 years. Notably, in previous driving simulation studies, this sample size was considered enough to arrive at some preliminary conclusions, especially using the EEG index. The number of participants used in previous studies has ranged from 10 to 24 (Reed and Green, 1999; Risser et al., 2000; Godley et al., 2002; Philip et al., 2005; Ingre et al., 2006).

### Driving Simulator and EEG System

**Figure 1:**
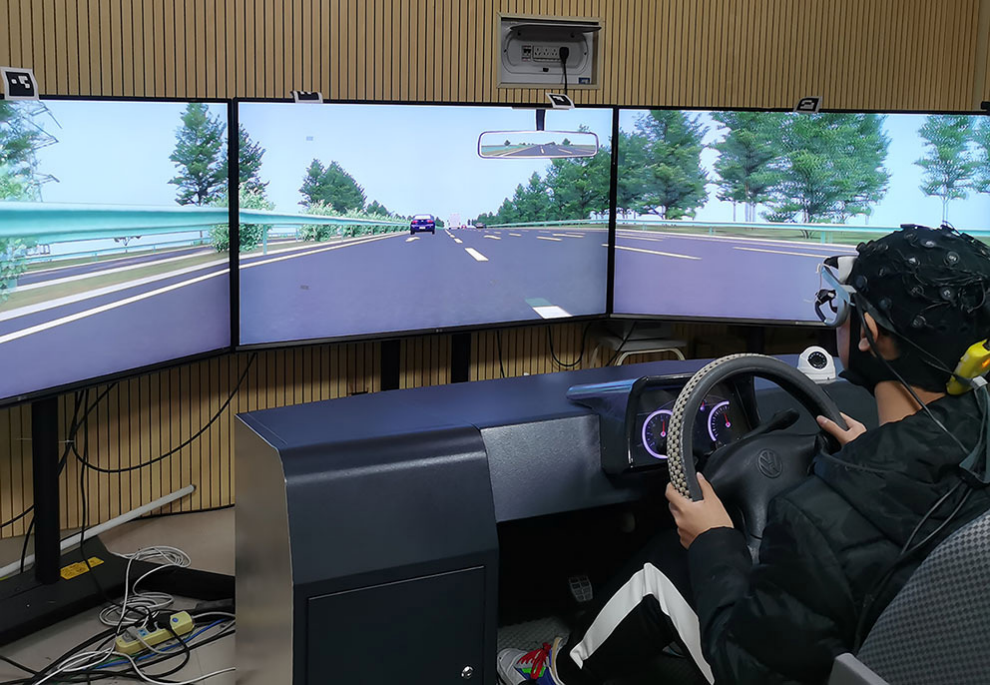
Experimental equipment.

The experiment was carried out indoor in a driving simulator cabin, eliminating the effects of weather, light, and noise on the experimental results. The indoor illumination was 300 lx. There is no noticeable light change in and out of the tunnel in the simulator test, which avoids physiological changes due to light changes, such as dim light that makes the pupils of drivers enlarged after entering the tunnel. The wireless EEG system and the driving simulator were used to collect the data in the experiments, as shown in Figure 1. The hardware parts of the driving simulator include the display, transmission, clutch, cockpit, sensors, steering, throttle, brake, and seat. The EEG acquisition part consists of the Enobio wireless EEG system, which transmits 24-bit EEG data and accurately restores the original EEG signal. Its bandwidth is 0–250 Hz, the sampling rate is 500 samples per second (sps), the resolution is 24 bits, i.e., 0.05 uv, and the noise is <1 uvrms (0–250 Hz).

The EEG test provides information concerning the dynamics and simulated electrical brain activity, where brain cells communicate through electrical impulses. An EEG can be used to help detect potential abnormalities in the brain waves. The small flat metal disks (i.e., electrodes) that are attached to the scalp with wires analyze the electrical impulses of the brain and send signals to a computer that records the results. The charges are amplified and appear as a graph on a computer screen. The details on the mathematical foundation of EEG can be found elsewhere (Doschoris and Kariotou, 2017).

### Experimental Scenarios

#### Experimental Road

**Figure 2:**
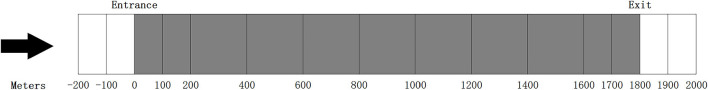
Schematic diagram of the experimental highway tunnel.

**Figure 3:**
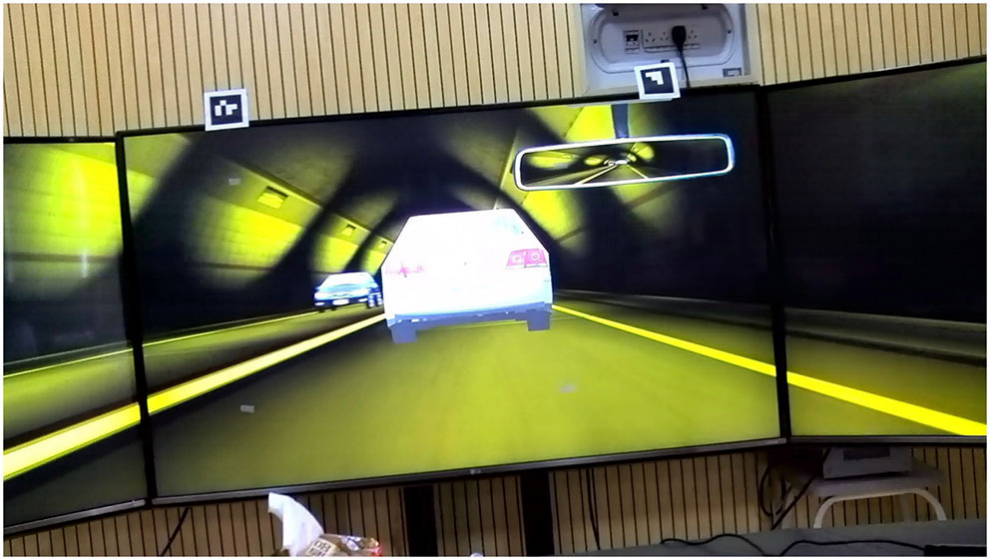
Schematic diagram of the internal environment of the tunnel.

The scene of this experiment was a long two-lane, second-class highway tunnel (i.e., one lane in each travel direction). According to the highway technical standards by the Standardization Administration of the People's Republic of China (Standardization Administration of the People's Republic of China, 2014), the length of the long highway tunnel ranges from 1,000 to 3,000 m. The tunnel length was set as 1,800 m, which is classified as a long tunnel. Also, this length would help control the total time of the experiment to avoid driver fatigue. The length of the experimental section was 2.2 km. Figure 2 shows a schematic diagram of the experimental highway tunnel. The length of the section from the starting point to the tunnel entrance was 200 m, and the length from the tunnel exit to the end point was 200 m. The width of each lane was 3.75 m, and the total width of the cross-section was 7.5 m (i.e., there was no median). Figure 3 shows a schematic diagram of the internal environment of the tunnel. A truck escape ramp existed on the right-hand side of the traveled way, with a width of 1.8 m. The experiment was carried out in the driving simulator, where the illumination was controlled as the indoor lighting (300 lx). There was no specific noise interfering with the participants, apart from the driving simulator sound.

#### Driving Speed and Traffic Flow

Both the mental state and the behavior of drivers were explored in a vehicle-following situation. The independent variable controlled in this study was a single factor, i.e., the speed of the leader vehicle. The speed limit of the second-class highway is normally set as 60 or 80 km/h, so this experiment took a design speed of 60 km/h as the maximum speed limit. The minimum speed limit zone was 30 km/h with increases of 15 km/h increments up to a maximum of 60 km/h. Thus, the three experimental scenarios in the following situations in the long tunnel were set with the driving speed of the front car set at 60 km/h (Scenario A), 45 km/h (Scenario B), and 30 km/h (Scenario C). Based on the United States Standards (Transportation Research Board, 2010), an average traffic flow in the steady-state of 700 passenger car units per hour (pcu/h) was selected as the experimental traffic flow for all scenarios.

### Experimental Process

Each subject needed to be familiar with the operation of the driving simulator before starting the formal experiment. The drivers followed the vehicle in front of them and drove according to their everyday driving habits. When driving in a tunnel, overtaking was forbidden. The experimental scenarios were based on a fully balanced method that eliminated the mutual interference of the testing sequence caused by various experimental conditions. Experiments were carried out in the morning, afternoon, and evening. The experiment was carried out in a room with curtains closed and lights on to reduce the influence of light. All participants were asked to have a good sleep before the experiment to ensure that they were full of energy and good mental state.

An example of the subject sequence was as follows: Subject 1 completed driving in Scenario A; after that, the driver rested for a while and then drove the simulator in Scenarios B and C. Then, Subject 2 would drive in the sequence of Scenarios C, A, and B, while Subject 3 drives in the sequence of Scenarios A, C, and B. The rest of the 21 subjects followed the same sequence pattern of the three scenarios. The EEG data, such as α-, β-, and θ-wave energy, were collected during the experiment. Thus, all 24 subjects participated in the investigation.

## Data Preprocessing

### Filtering and Re-referencing

The filtering mainly filtered the useless part of the original EEG data from 32 electrode locations and included two steps. The first step was the low-pass filtering, which filtered the EEG waveform with a frequency below 0.5 Hz. The second step was the high-pass filtering, which screened the EEG waveform with a frequency above 40 Hz (i.e., this frequency is commonly used for EEG pre-analysis). The re-referencing is to use the average value of EEG data on each electrode as a reference for calibration to prevent the artifacts from affecting the overall EEG data.

### Independent Component Analysis Technology Denoising

The independent component analysis (ICA) is a data processing technology widely used in many fields. The ICA technology is mainly used to find and remove specific EEG artifacts after filtering and re-referencing in EEGLAB.

### EEG Energy Value Data Extraction

**Figure 4:**
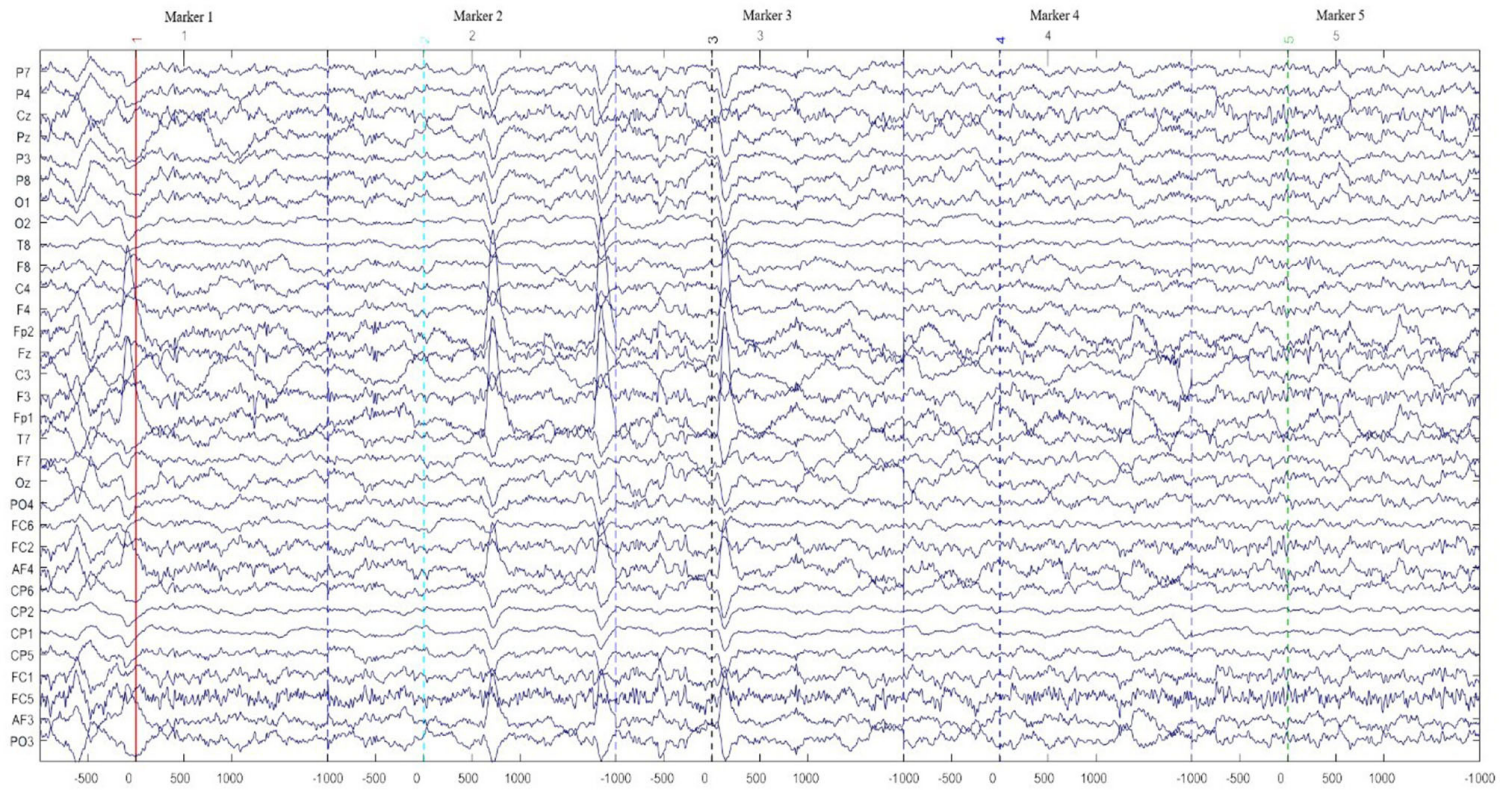
Sample electroencephalogram (EEG) waveforms after removing artifacts.

In this experiment, the data for the EEG energy value were extracted in the α, β, and θ waves, and the wave ratio (α + θ)/β. Their characteristics are presented in Table 1. The specific extraction process was as follows:

- Export the original data from the Enobio system and import it into the EEGLAB toolbox. Perform filtering, sequencing, and ICA preprocessing to remove unwanted frequency waves and noise.
- Use EEGLAB to remove the artifacts that still existed manually. The EEG signal after removing the artifacts is shown in Figure 4. Y-axis represents the 32 channels of EEG. The numbers −500, 0, 500, and 1,000 in the X-axis represent −500, 0, 500, and 1,000 ms at this marker time point. The changing lines in the figure represent the change of EEG collected from the participants. The marker 1–5 at the top of the figure refers to the time when the EEG data were collected from the participants. The markers are the specific points used to collect EEG signals in the experiment. The other six markers are not shown in the figure due to the size of the window. The markers are set every 300 m to collect the data of drivers for the first 500 ms and then 1,000 ms, adding up to 11 markers in this experiment. Then, MATLAB was used to extract the EEG energy data (μv^2^), which correspond to various frequency waves, into Excel files for analysis.

**Table 1:** Characteristics of three types of electroencephalogram (EEG) rhythms and a composite index.

**EEG wave**	**Frequency (Hz)**	**Cognitive characteristics**
α	8–13	A low-amplitude synchronous wave. The main waveform is recorded in the awake and quiet state. It is generally considered to be related to the preparation of the brain.
β	13–30	A high-frequency, low-amplitude asynchronous fast wave. It reflects the alertness state of the brain, which can be seen when nervous or excited, indicating that the cerebral cortex is in an excited state.
θ	4–8	It belongs to the low-to-medium amplitude slow wave, which appears when people calmly relax and turn to sleep. It manifests the central nervous system's inhibitory state and is related to working memory load.
Wave ratio	-^a^	An EEG composite index related to mental fatigue, suggesting the higher ratio, the higher the mental fatigue level.

a *Not applicable*.

## Results and Discussion

### Analysis of Repeated Measurement of Variance

This analysis was based on the mean values of different speeds and involved Mauchly's sphericity test and the within-subject test. Mauchly's spherical hypothesis is used to test the autocorrelation degree of each level in a group. The repeated measures ANOVA is based on Mauchly's sphericity test of each data. If the spherical assumption is not satisfied, the Epsilon test should be used to correct the error. According to Tables 2, 3, the significance of all variables was >0.05, which satisfied the spherical assumption, and thus, the Epsilon correction was not required.

**Table 2:** Mauchly's test of sphericity.

**Variable (in frontal lobe)**	**Mauchly's W**	**Approx. Chi-Square**	**df**	**Sig**.	**Epsilon Greenhouse-Geisser**
α-wave energy	0.785	2.423	2	0.298	0.823
β-wave energy	0.935	0.667	2	0.716	0.939
θ-wave energy	0.930	0.730	2	0.694	0.934
Wave ratio	0.659	4.170	2	0.124	0.746

**Table 3:** Tests of within-subject effects.

**Variable (in frontal lobe)**	**Inspection type assumed**	**Type III sum of squares**	**Df**	**Mean squares**	***F***	**Sig**.	**Partial eta squared**
α-wave energy	Sphericity	610055.9	2	305027.9	0.705	0.505	0.060
β-wave energy	Sphericity	1507640.6	2	753820.3	2.722	0.088	0.198
θ-wave energy	Sphericity	2307488.3	2	1153744.1	0.127	0.881	0.011
Wave ratio	Sphericity	0.898	2	0.449	1.865	0.179	0.145

The within-subject test aims to study the relationship between each dependent variable and the speed change. The within-subject tests refer to the comparison of the mean-variance among the groups. There would be no significant difference in the mean among the groups if *p* > 0.05. If the statistical significance requirement is met, this proves that the speed change has a substantial effect on the dependent variable. All the indicators meet the spherical assumption. However, in terms of the speed of the assumed sphericity, only the β-wave and the wave ratio are rather significant but are still not <0.05. The indifference assumption is accepted. Although all variables in the within-subject effects were not significant, in the tests of the within-subject contrasts shown in Table 4, the significance of the β-wave energy and the wave ratio in the second measurement were 0.037 and 0.031, respectively, with a *p*-value < 0.05. As noted, under the quadratic relationship, the β-wave energy and the wave ratio are related to the speed effect.

**Table 4:** Tests of within-subject contrasts.

**Variable (in frontal lobe)**	**Speed**	**Type III sum of squares**	**Df**	**Mean square**	***F***	**Sig**.	**Partial eta squared**
α-wave energy	Linear	252919.3	1	252919.3	0.457	0.513	0.040
	Quadratic	357136.6	1	357136.6	1.146	0.307	0.094
β-wave energy	Linear	213979.4	1	213979.4	0.663	0.433	0.057
	Quadratic	1293661.2	1	1293661.2	5.593	0.037	0.337
θ-wave energy	Linear	166878.0	1	166878.0	0.021	0.887	0.002
	Quadratic	2140610.2	1	2140610.2	0.210	0.656	0.019
Wave ratio	Linear	0.013	1	0.013	0.038	0.848	0.003
	Quadratic	0.885	1	0.885	6.102	0.031	0.357

### EEG Test Comparison During Driving

This test evaluates the significance of the changes in the EEG data during the follow-up process under a car-following situation. This test would help indicate whether the EEG data have changed significantly during the entire driving process. When the distance is 0, 100, 400, 600, 800, 1,200, 1,400, and 1,600 m, the number of contrasts is ≥2, and the significance of each indicator is ≤0.05, indicating that the energy value indicators are changed significantly at these positions. In general, as the driving distance changes, the changes in the EEG energy value are statistically significant. Table 5 presents the results of the tests of contrasts of the three EEG indicators, which show the significance of the change in these indicators during the driving process.

**Table 5:** Tests of contrasts of different speeds for various driving distances.

**Variable (in frontal lobe)**	**Speed (km/h)**	**Comparison distance (m)**		**Sig**.
		**First distance**	**Second distance**	
α-wave	60	100	400	0.049
α-wave	60	400	600	0.048
α-wave	30	100	1,000	0.033
θ-wave	45	0	400	0.019
θ-wave	45	100	400	0.021
θ-wave	45	200	400	0.040
θ-wave	45	400	600	0.008
θ-wave	45	400	1,200	0.017
θ-wave	45	400	1,600	0.012
θ-wave	45	400	1,800	0.016
θ-wave	30	0	1,200	0.042
θ-wave	30	100	400	0.034
θ-wave	30	100	1,000	0.038
θ-wave	30	100	1,200	0.003
θ-wave	30	100	1,600	0.033
θ-wave	30	400	800	0.041
θ-wave	30	600	1,200	0.023
θ-wave	30	800	1,000	0.048
θ-wave	30	800	1,200	0.002
θ-wave	30	800	1,600	0.041
θ-wave	30	1,200	1,400	0.014
θ-wave	30	1,200	1,700	0.025
(α + θ) / β	30	0	1,200	0.034
(α + θ) / β	30	100	1,200	0.014
(α + θ) / β	30	800	1,200	0.037
(α + θ) / β	30	1,200	1,400	0.008
(α + θ) / β	30	1,200	1,600	0.036

### EEG Topography in Three Following Scenarios in the Tunnel

**Figure 5:**
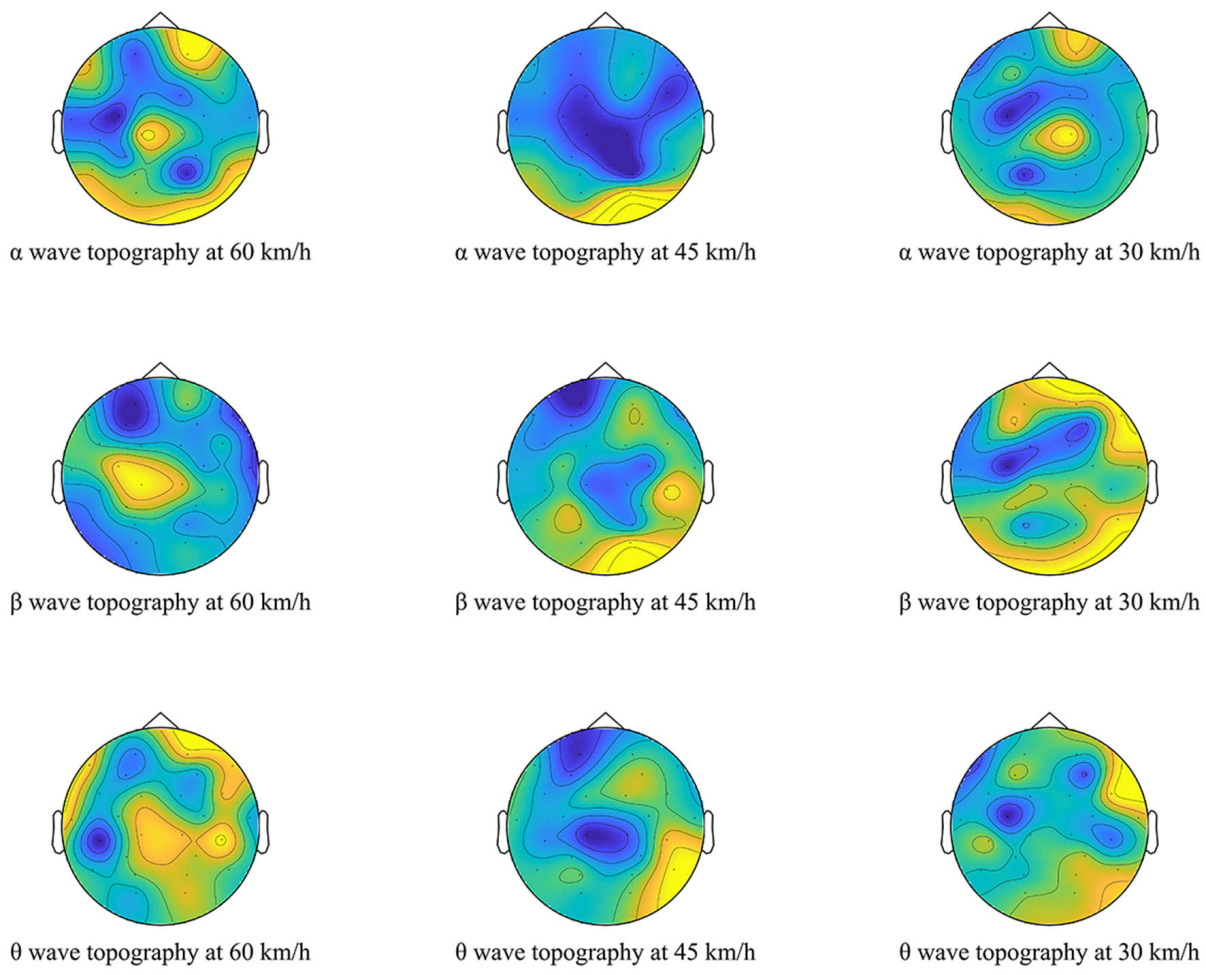
EEG topographic maps of the three waves of drivers for different speeds.

Figure 5 shows the EEG topographic maps of the three waves for different speeds. The color in the topographic map (from yellow to dark blue) indicates the activation level of the brain wave. The bluer the color, the less active it is. Intuitively, under the scenarios of the α-wave and β-wave at 45 km/h, the blue area of the EEG topographic map is larger than that at 30 and 60 km/h, indicating that the mental state of drivers at 45 km/h is more stable than at other speeds. Notably, the depth of the color in the map represents the visualized EEG activity, which directly reflects the activity of drivers at each following speed. However, the map cannot be used for the quantitative analysis.

### Change in EEG Energy During Driving

The statistical analysis of the change in EEG shows the significance of the β-wave energy and the wave ratio. The second measurements are 0.037 and 0.031, respectively, with *p*-value < 0.05. These results indicate that driving psychology varies for different vehicle-following conditions in the tunnel. The changes of the four EEG indicators during the driving process are described in the following. The unit of EEG wave energy is μv^2^, which represents the intensity of the three brain waves.

#### α-Wave Energy in the Frontal Lobe

**Figure 6:**
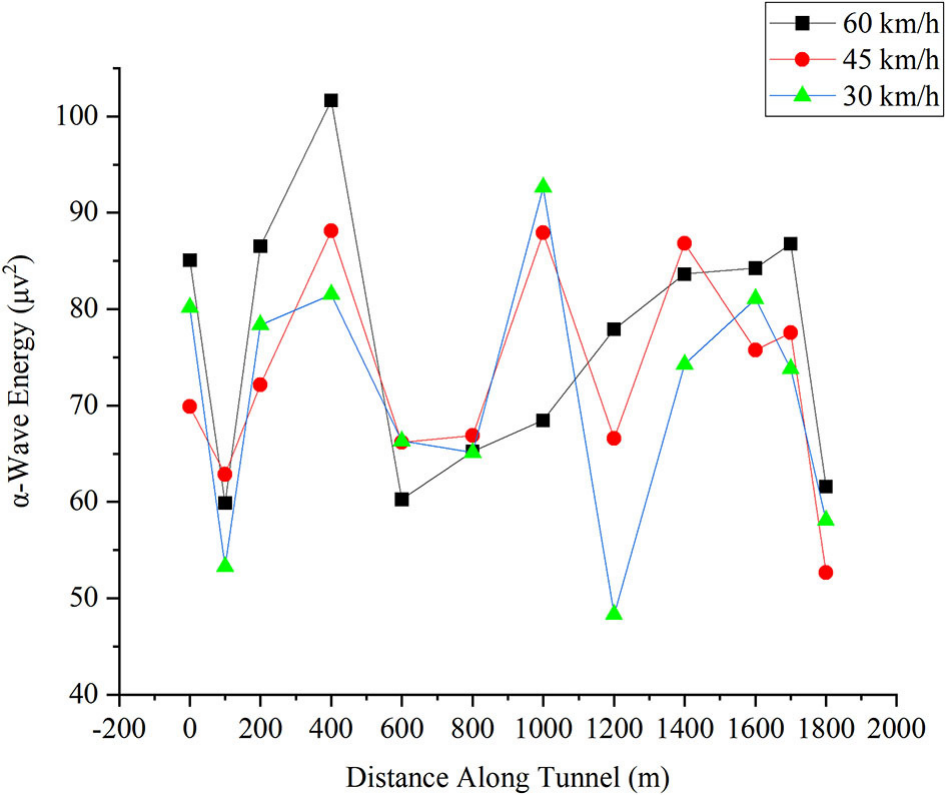
Change in α-wave energy in the frontal lobe for different driving speeds.

The change in the α-wave energy in the frontal lobe for different driving speeds is shown in Figure 6. The X-axis is the distance the driver reached in the tunnel, and the Y-axis is the magnitude of the α-wave energy. As noted, as the driver drives in the tunnel, the energy value of the α-wave of the EEG decreases when the driver enters the first 100 m. When the α-wave energy decreases, the drowsiness of the driver is reduced, and the cognitive resources increase (Schier, 2000). This corresponds to the concentration of attention when entering the tunnel (Klimesch et al., 1998; Wang et al., 2015). As the driving distance increases, the α-wave energy increases two times and also decreases two times. The first increase occurs when the driver enters the middle of the tunnel, reaching the highest point at 400 m, and during 600–800 m, the α-wave energy fell rapidly. This trend indicates the distracted and concentrated process of drivers (from distraction to concentration). Then, during the driving process, the α-wave energy increases, but it does not recover to the level at 400 m. This trend might be due to that the driver is reaching the exit of the tunnel, where the driver is relieved when driving out of the tunnel without the tension of following other cars in the tunnel. However, the driver is still concentrating along with the highway driving conditions. After the driver enters the tunnel environment, the adaptation to the tunnel environment and the process of focusing attention have kept his level of drowsiness at a low level. As the driver enters the exit of the tunnel at 1,800 m, the driving task ends. The attention level of drivers drops rapidly.

#### β-Wave Energy in the Frontal Lobe

**Figure 7:**
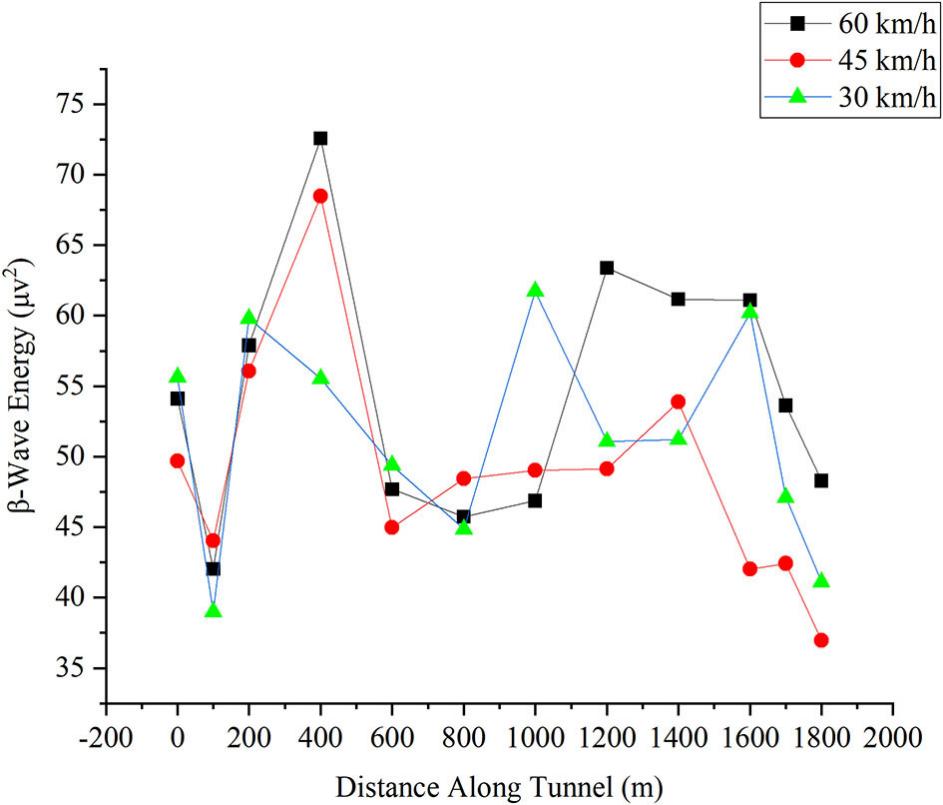
Change in β-wave energy in the frontal lobe for different driving speeds.

The change in the β-wave energy in the frontal lobe for different driving speeds is shown in Figure 7. The X-axis is the distance the driver reached in the tunnel, and the Y-axis is the magnitude of the β-wave energy. As noted, the β-wave energy still shows the same effect as the participants drove in the first 0–400 m, i.e., the decline-increasing process when entering the tunnel. The increase in the β-wave represents an increase in the alertness level (Eoh et al., 2005). When the driver enters the tunnel, the alertness level first decreases and then increases. This reveals a distractive pattern of the drivers who enter the tunnel. In the first 100 m, drivers are distracted (due to the pressure of the tunnel, the environment, or the changing environment). However, in the next 100 m, the drivers regain their alertness when they adapt to the tunnel environment. After 400 m, the β*-*wave energy (i.e., the alertness level of drivers) is the lowest at 45 km/h. Then, from 800 to 1,600 m, the β*-*wave energy at 45 km/h is decreased steadily in contrast to the oscillation of the other two brain wave energies at the other speeds. This indicates that after adapting to the tunnel environment, the driver is more comfortable at 45 km/h.

#### θ-Wave Energy in the Frontal Lobe

**Figure 8:**
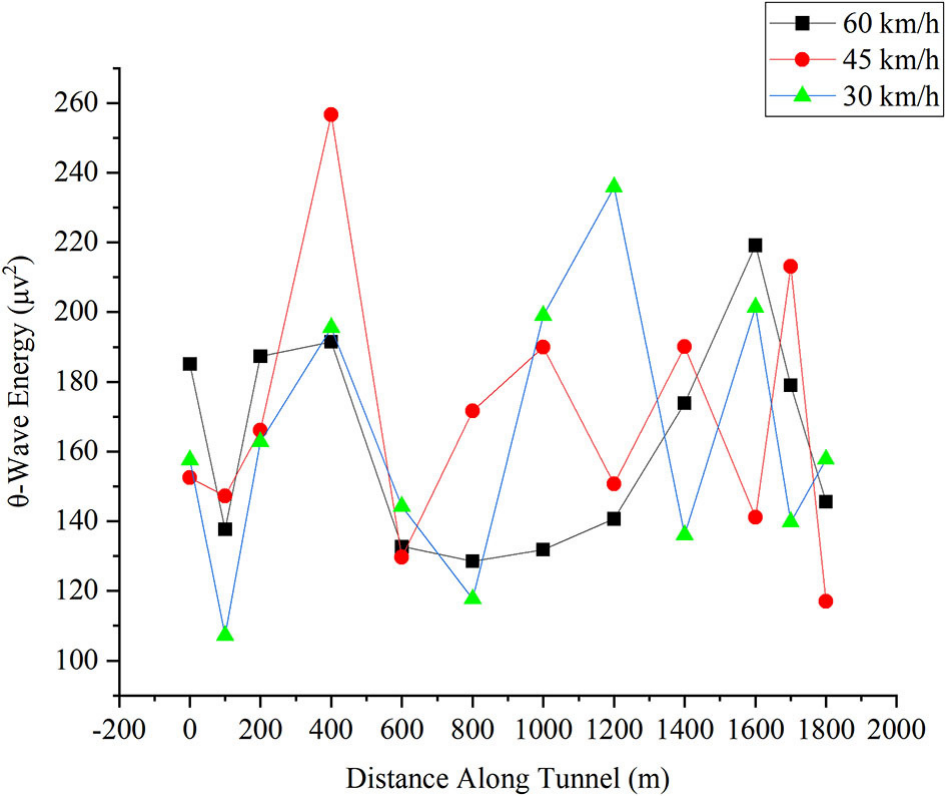
Change in θ-wave energy in the frontal lobe for different driving speeds.

Figure 8 shows the change in the θ-wave energy in the frontal lobe for different driving. The θ-wave energy does not show significance in the repeated measures ANOVA. The X-axis is the distance the driver reached in the tunnel. The Y-axis is the magnitude of θ-wave energy. Its unit is μv^2^. In the contrast tests, compared with the energy of the other two speeds, the θ-wave energy value at 30 km/h had changed significantly between most of the distances. At the three speeds, the θ-wave energy value of drivers still maintained a descent-rise at 400 m at the tunnel entrance, but at 45 km/h, the fall is not apparent. At 400 m, the θ-wave energy is higher than the other two speeds. Because the driver alertness level decreased when the θ-wave energy increased (Åkerstedt et al., 1991), the driver alertness decreased at the entrance. As noted, the θ-wave energy for 30 km/h is higher than that for the other two speeds during the distance from 800 to 1,400 m, but after that, the θ-wave energy is less than that for the other speeds. This trend is similar to the α-wave energy where the θ-wave energy at 45 km/h does not recover to the level of 400 m. This might also be due to the relief of drivers, as noted in the analysis of the α-wave energy. At 60 km/h, the θ-wave energy also has a descent-rise, presumably indicating that the driver is attentive to the tunnel entrance.

#### Wave Ratio in the Frontal Lobe

**Figure 9:**
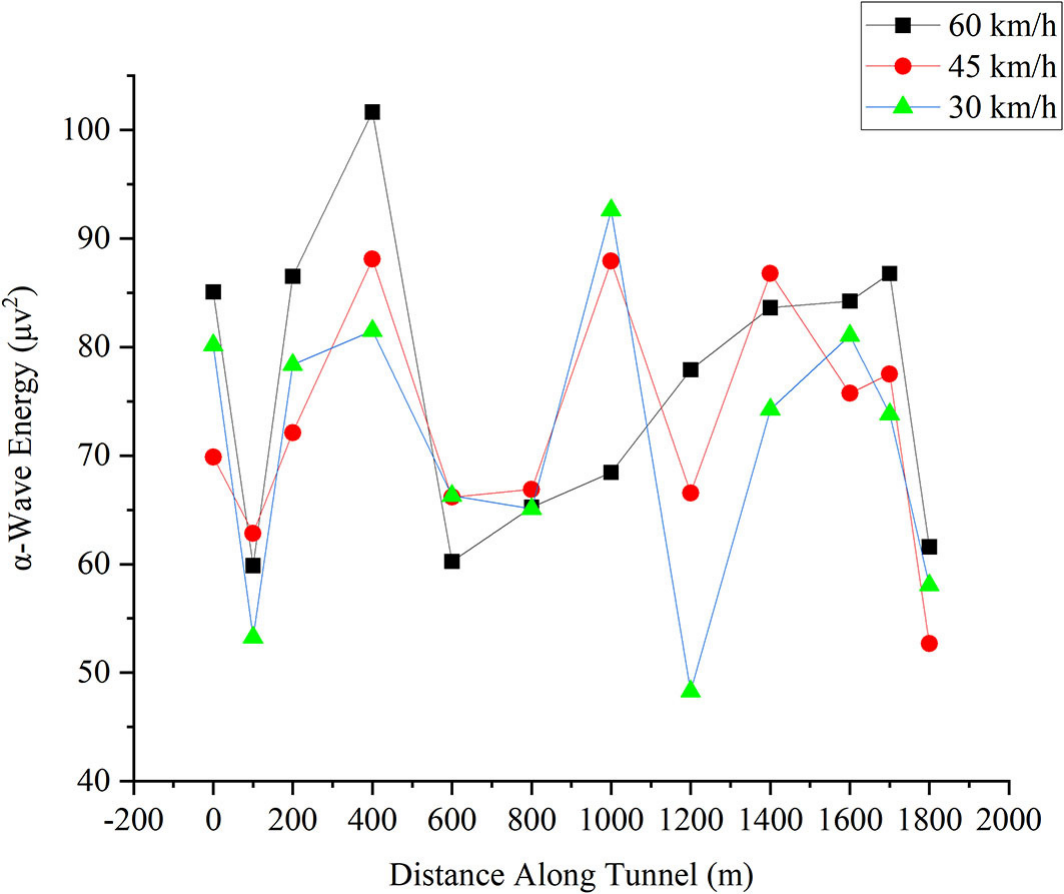
Change in the wave ratio in the frontal lobe for different driving speeds.

The wave ratio has been demonstrated in the repeated measures ANOVA to illustrate the significance of change with speed. Figure 9 shows the change in the wave ratio in the frontal lobe for different driving speeds. The X-axis is the distance the driver reached in the tunnel, and the Y-axis is the magnitude of the wave ratio. As noted, the drop-rise effect occurred during 0–200 m. From 0 to 600 m, the ratio of the three following car speeds is almost the same, but at 600 m, the ratio at 45 km/h started to rise, drops at a distance between 1,200 and 1,600 m, and then sharply rises at 1,700 m. After the jump, the ratio decreases again. After 1,200 m, the ratio for 45 km/h is in the neutral position relative to the other two speeds (although a sudden jump occurs at 1,700 m) and then returns to around 5 at the exit. The increase in the wave ratio as driver fatigue increases (Cao et al., 2014) indicates that the fatigue value for 45 km/h is generally in the neutral position during tunnel driving. The ratio is not much different from that of the other two speeds.

## Conclusion

This study has evaluated three simulated driving scenarios in a long highway tunnel with different speeds of the front vehicle. The EEG technology was used to provide information about the dynamics and simulated electrical brain activity of subjects. Based on this study, the following comments are offered:

1. The analysis of the EEG energy data showed that the driver had the lowest alertness level and the best driving experience at 45 km/h. The wave ratio showed that the fatigue value for 45 km/h was generally in the middle and was not much different from the other two speeds. The study experiments involved a specific two-lane tunnel (i.e., one lane in each direction), which is 1,800 m long, and participants with an age range of 20–30 years. Since the tunnel length and the age of participants would affect the optimum speed of the tunnel, the preliminary finding that the 45 km/h is an appropriate speed applies only to the two-lane highway tunnels with lighting/sound and participants similar to those used in this study. Also, this finding is likely to apply to the tunnels in China, which exhibit similar conditions and may not be transferrable to other countries.
2. The results showed that the EEG energy of drivers had a falling–rising effect during tunnel driving. Since the environmental illuminance of the experiment was controlled, there was no interference in the dark adaptation process in vision. The analysis of the α, β, and θ waves revealed a similar effect in the EEG index when driving in the middle and the rear end of the tunnel. Researchers have recently studied the sustained attention and the attention behavior in distracted driving (Wang et al., 2013; Pallavi and Harish, 2016; Ding et al., 2019; Karran et al., 2019), especially the reduction and oscillations of the α*-*wave energy and the increase in the θ*-*wave energy (Klimesch et al., 1998; Wang et al., 2015). It could be speculated that the trends of these wave energies were related to the attention–concentration behavior of drivers during tunnel driving.
3. The results of the study showed different psychological reactions of drivers at different speeds. For the three speeds of 60, 45, and 30 km/h, the drivers had a better driving experience at a moderate speed (i.e., 45 km/h) when following the front car. A falling-rising effect on the three brain waves was also found. This finding might contribute to the analysis of driving behavior in the tunnel using physiological indicators. Furthermore, the conclusion of this study may help set the index weight when modeling the car-following behavior from the psychological state of drivers. Also, the finding might strengthen the inference and analysis of the motivation and mechanism of lousy behavior of drivers and might provide a theoretical basis for the prevention of tunnel traffic accidents.
4. This study has some limitations. The error of the EEG data process was relatively large, and the test scene was too restrictive. In addition, it was necessary to study the speed variable only due to the experimental conditions. Future studies might consider more complex driving scenarios involving a broader selection of speeds and various physiological indicators, such as ECG and skin electricity, which can be combined to study driver behavior more comprehensively. In addition, the effect of different tunnel lighting and noise levels can be explored.

## Data Availability Statement

The original contributions presented in the study are included in the article/supplementary material, further inquiries can be directed to the corresponding author/s.

## Ethics Statement

The studies involving human participants were reviewed and approved by Committee of Department of Civil Engineering, Fuzhou University. The patients/participants provided their written informed consent to participate in this study. Written informed consent was obtained from the individual(s) for the publication of any potentially identifiable images or data included in this article.

## Author Contributions

YY and YF contributed to the study conception and design. YF performed the data collection. YY, YF, XZ, and SE contributed to the analysis and interpretation of results. YF, XZ, and SE prepared and drafted the manuscript. All authors reviewed the results and approved the final version of the manuscript.

## Funding

This study was financially supported by the Traffic Research Center and College of Civil Engineering of Fuzhou University, the Fujian Provincial Department of Transportation, the Hebei Provincial Department of Transportation, and the Fujian Province Young and Middle-aged Teacher Education and Research Project (JT180852), China.

## Conflict of Interest

The authors declare that the research was conducted in the absence of any commercial or financial relationships that could be construed as a potential conflict of interest.

## Publisher's Note

All claims expressed in this article are solely those of the authors and do not necessarily represent those of their affiliated organizations, or those of the publisher, the editors and the reviewers. Any product that may be evaluated in this article, or claim that may be made by its manufacturer, is not guaranteed or endorsed by the publisher.

## References

[B1] Åkerstedt T. Kecklund G. Knutsson A. (1991). Manifest sleepiness and the spectral content of the EEG during shift work. Sleep 14, 221–225. 10.1093/sleep/14.3.221 1896723

[B2] Amundsen F. H. Ranes G. (2000). Studies on traffic accidents in Norwegian road tunnels. Tunnel. Undergr. Space Technol. Incorporat. Trenchless Technol. Res. 15, 3–11. 10.1016/S0886-7798(00)00024-9

[B3] Arakawa T. Hibi R. Fujishiro T. A. (2019). Psychophysical assessment of a driver's mental state in autonomous vehicles. Transport. Res. A Pol. Pract. ce 124, 587–610. 10.1016/j.tra.2018.05.003

[B4] Barua S. Ahmed M. U. Begum S. (2017). Classifying drivers' cognitive load using EEG signals. Stud. Health Technol. Inform. 237, 99–106. 28479551

[B5] Bashivan P. Rish I. Heisig S. (2016). Mental State Recognition via Wearable EEG. Available online at: https://export.arxiv.org/abs/1602.00985 (accessed June 05, 2016).

[B6] Bassan S. (2016). Overview of traffic safety aspects and design in road tunnels. IATSS Res. 40, 35–46. 10.1016/j.iatssr.2016.02.002

[B7] Borghini G. Vecchiato G. Toppi J. Astolfi L. Maglione A. Isabella R.. (2012). Assessment of mental fatigue during car driving by using high resolution EEG activity and neurophysiologic indices, in 2012 Annual International Conference of the IEEE Engineering in Medicine and Biology Society, (San Diego, CA: IEEE), 6442–6445. 10.1109/EMBC.2012.6347469 23367404

[B8] Caliendo C. De Guglielmo M. L. (2012). Accident rates in road tunnels and social cost evaluation. Proc. Soc. Behav. Sci. 53, 166–177. 10.1016/j.sbspro.2012.09.870

[B9] Calvi A. D'amico F. (2013). A study of the effects of road tunnel on driver behavior and road safety using driving simulator. Adv. Transport. Stud. 30, 59–76.

[B10] Cao T. Wan F. Wong C. da Cruz J. Hu Y. (2014). Objective evaluation of fatigue by EEG spectral analysis in steady-state visual evoked potential-based brain-computer interfaces. Biomed. Eng. Onl. 13:28. 10.1186/1475-925X-13-28 24621009 PMC3995691

[B11] Ding S. Yuan Z. An P. Xue G. Sun W. Zhao J. (2019). Cascaded convolutional neural network with attention mechanism for mobile EEG-based driver drowsiness detection system, in 2019 IEEE International Conference on Bioinformatics and Biomedicine (BIBM) (San Diego, CA: IEEE), 1457–1464. 10.1109/BIBM47256.2019.8982938

[B12] Doschoris M. Kariotou F. (2017). Mathematical foundation of electroencephalography, in Electroencephalography, ed Sittiprapaporn P. (London: IntechOpen). Available online at: https://www.intechopen.com/chapters/54651

[B13] Eoh H. J. Chung M. K. Kim S.-H. (2005). Electroencephalographic study of drowsiness in simulated driving with sleep deprivation. Int. J. Indus. Ergon. 35, 307–320. 10.1016/j.ergon.2004.09.006

[B14] Fan X.-A. Bi L.-Z. Chen Z.-L. (2010). Using EEG to detect drivers' emotion with Bayesian Networks, in 2010 International Conference on Machine Learning and Cybernetics (Qingdao: IEEE), 1177–1181. 10.1109/ICMLC.2010.5580919

[B15] Godley S. T. Triggs T. J. Fildes B. N. (2002). Driving simulator validation for speed research. Accid. Anal. Prev. 34, 589–600. 10.1016/S0001-4575(01)00056-2 12214953

[B16] Haak M. Bos S. Panic S. Rothkrantz L. (2008). Detecting stress using eye blinks and brain activity from EEG signals, in Proceeding of the 1st Driver Car Interaction and Interface (DCII 2008). Prague.

[B17] Ingre M. Åkerstedt T. Peters B. Anund A. Kecklund G. (2006). Subjective sleepiness, simulated driving performance and blink duration: examining individual differences. J. Sleep Res. 15, 47–53. 10.1111/j.1365-2869.2006.00504.x 16490002

[B18] Jap B. T. Lal S. Fischer P. Bekiaris E. (2009). Using EEG spectral components to assess algorithms for detecting fatigue. Expert Syst. Appl. 36, 2352–2359. 10.1016/j.eswa.2007.12.043

[B19] Karran A. J. Demazure T. Leger P.-M. Labonte-LeMoyne E. Senecal S. Fredette M.. (2019). Toward a hybrid passive BCI for the modulation of sustained attention using EEG and fNIRS. Front. Hum. Neurosci. 13:393. 10.3389/fnhum.2019.00393 31780914 PMC6851201

[B20] Kim H. S. Yoon D. Shin H. S. Park C. H. (2018). Predicting the EEG level of a driver based on driving information. IEEE Trans. Intell. Transport. Syst. 20, 1215–1225. 10.1109/TITS.2018.2848300

[B21] Kinateder M. Pauli P. Müller M. Krieger J. Heimbecher F. Rönnau I.. (2013). Human behaviour in severe tunnel accidents: effects of information and behavioural training. Transport. Res. F Traffic Psychol. Behav. 17, 20–32. 10.1016/j.trf.2012.09.001

[B22] Kiymik M. K. Akin M. Subasi A. (2004). Automatic recognition of alertness level by using wavelet transform and artificial neural network. J. Neurosci. Methods 139, 231–240. 10.1016/j.jneumeth.2004.04.027 15488236

[B23] Klimesch W. Doppelmayr M. Russegger H. Pachinger T. Schwaiger J. (1998). Induced alpha band power changes in the human EEG and attention. Neurosci. Lett. 244, 73–76. 10.1016/S0304-3940(98)00122-0 9572588

[B24] Lemke M. (1982). Correlation between EEG and driver's actions during prolonged driving under monotonous conditions. Accid. Anal. Prev. 14, 7–17. 10.1016/0001-4575(82)90003-3

[B25] Li M.-a. Zhang C. Yang J.-F. (2010). An EEG-based method for detecting drowsy driving state, in 2010 Seventh International Conference on Fuzzy Systems and Knowledge Discovery (Yantai: IEEE), 2164–2167. 10.1109/FSKD.2010.5569757

[B26] Lin C.-T. Chuang C.-H. Huang C.-S. Tsai S.-F. Lu S.-W. Chen Y.-H.. (2014). Wireless and wearable EEG system for evaluating driver vigilance. IEEE Trans. Biomed. Circ. Syst. 8, 165–176. 10.1109/TBCAS.2014.2316224 24860041

[B27] Lin C.-T. Wu R.-C. Liang S.-F. Chao W.-H. Chen Y.-J. Jung T.-P. (2005). EEG-based drowsiness estimation for safety driving using independent component analysis. IEEE Trans. Circ. Syst. I Reg. Pap. 52, 2726–2738. 10.1109/TCSI.2005.857555

[B28] Liu N.-H. Chiang C.-Y. Hsu H.-M. (2013). Improving driver alertness through music selection using a mobile EEG to detect brainwaves. Sensors 13, 8199–8221. 10.3390/s130708199 23803789 PMC3758591

[B29] Ma Z.-l. Shao C.-f. Zhang S.-r. (2009). Characteristics of traffic accidents in Chinese freeway tunnels. Tunnel. Undergr. Space Technol. 24, 350–355. 10.1016/j.tust.2008.08.004

[B30] Miyake K. Ota S. Shigematsu D. Ohkusa K. Ikeda Y. Jinno M. (2019). Visibility improvement in expressway tunnels by optimizing the color temperature and light distribution of the pulse-operated LED luminaires. J. Sci. Technol. Light. 42, 22–28. 10.2150/jstl.IEIJ170000620

[B31] Pallavi T. Harish G. (2016). Implementation of EEG based driver's attention tracking and habitats monitoring system, in 2016 International Conference on Communication and Electronics Systems (ICCES) (Coimbatore: IEEE), 1–4. 10.1109/CESYS.2016.7889852

[B32] Philip P. Sagaspe P. Taillard J. Valtat C. Moore N. Åkerstedt T.. (2005). Fatigue, sleepiness, and performance in simulated versus real driving conditions. Sleep 28, 1511–1516. 10.1093/sleep/28.12.1511 16408409

[B33] Reed M. P. Green P. A. (1999). Comparison of driving performance on-road and in a low-cost simulator using a concurrent telephone dialling task. Ergonomics 42, 1015–1037. 10.1080/001401399185117

[B34] Resalat S. N. Saba V. (2015). A practical method for driver sleepiness detection by processing the EEG signals stimulated with external flickering light. Sign. Image Video Proces. 9, 1751–1757. 10.1007/s11760-015-0760-x

[B35] Risser M. R. Catesby W. J. Freeman F. G. (2000). Driving simulation with EEG monitoring in normal and obstructive sleep apnea patients. Sleep 3:393. 10811383

[B36] Schier M. A. (2000). Changes in EEG alpha power during simulated driving: a demonstration. Int. J. Psychophysiol. 37, 155–162. 10.1016/S0167-8760(00)00079-9 10832002

[B37] Standardization Administration of the People's Republic of China (2014). China Highway & Transportation Standards: Technical Standard of Highway Engineering JTG-B01-2014. Beijing.

[B38] Torsvall L. (1987). Sleepiness on the job: continuously measured EEG changes in train drivers. Electroencephal. Clin. Neurophysiol. 66, 502–511. 10.1016/0013-4694(87)90096-4 2438115

[B39] Transportation Research Board (2010). Highway Capacity Manual 2010. Washington, DC: TRB, National Research Council.

[B40] Wang Y.-K. Jung T.-P. Chen S.-A. Huang C.-S. Lin C.-T. (2013). Tracking attention based on EEG spectrum, in HCI International 2013 - Posters' Extended Abstracts. HCI 2013. Communications in Computer and Information Science, vol 373, ed. S. C. (Berlin, Heidelberg: Springer), 450–454. 10.1007/978-3-642-39473-7_90

[B41] Wang Y. K. Jung T. P. Lin C. T. (2015). EEG-based attention tracking during distracted driving. IEEE Trans. Neural Syst. Rehabil. Eng. 23, 1085–1094. 10.1109/TNSRE.2015.2415520 25850090

[B42] Yan Y. Dai Y. Li X. Tang J. Zhongyin G. (2019). Driving risk assessment using driving behavior data under continuous tunnel environment. Traffic Injury Prev. 20, 807–812. 10.1080/15389588.2019.1675154 31738591

[B43] Yang L. Guan W. Ma R. Li X. (2019a). Comparison among driving state prediction models for car-following condition based on EEG and driving features. Accid. Anal. Prev. 133:105296. 10.1016/j.aap.2019.105296 31563015

[B44] Yang L. Ma R. Zhang H. M. Guan W. Jiang S. (2018). Driving behavior recognition using EEG data from a simulated car-following experiment. Accid. Anal. Prev. 116, 30–40. 10.1016/j.aap.2017.11.010 29174606

[B45] Yang Y. Chen J. Easa S. Yu S. Zheng X. (2020a). Effectiveness of yellow color guardrail belt at freeway exits. Accid. Anal. Prev. 146:105737. 10.1016/j.aap.2020.105737 32882616

[B46] Yang Y. Chen M. Wu C. Easa S. M. Zheng X. (2020b). Structural equation modeling of drivers' situation awareness considering road and driver factors. Front. Psychol. 11:1601. 10.3389/fpsyg.2020.01601 32793039 PMC7385403

[B47] Yang Y. Chen Y. Wu C. Easa S. M. Lin W. Zheng X. (2020c). Effect of highway directional signs on driver mental workload and behavior using eye movement and brain wave. Accid. Anal. Prev. 146:105705. 10.1016/j.aap.2020.105705 32818759

[B48] Yang Y. Easa S. M. Zheng X. Hu A. Liu F. (2019b). Evaluation effects of two types of freeway deceleration markings in China. PLoS ONE 14:e0220811. 10.1371/journal.pone.0220811 31408464 PMC6692039

[B49] Yang Y. Jianying C. Said M. Easa Xinyi Z. Wei L. Yichuan P. (2020d). Driving simulator study of the comparative effectiveness of monolingual and bilingual guide signs on Chinese highways. Transport. Res. F Traffic Psychol. Behav. 68, 67–78. 10.1016/j.trf.2019.11.008

[B50] Zeng H. Yang C. Dai G. Qin F. Zhang J. Kong W. (2018). EEG classification of driver mental states by deep learning. Cogn. Neurodyn. 12, 597–606. 10.1007/s11571-018-9496-y 30483367 PMC6233328

